# Successful treatment of acrodermatitis continua of Hallopeau coexisting with generalized pustular psoriasis with spesolimab: a case report

**DOI:** 10.3389/fimmu.2024.1338285

**Published:** 2024-02-23

**Authors:** Pengfei Wen, Chuan Liu, Tingting Wang, Xian Jiang, Ping Wang, Sheng Wang

**Affiliations:** ^1^ Department of Dermatovenerology, West China Hospital, Sichuan University, Chengdu, China; ^2^ The Department of Dermatology, First Affiliated Hospital of Chongqing Medical University, Chongqing, China

**Keywords:** generalized pustular psoriasis, acrodermatitis continua of Hallopeau, spesolimab, treatment, IL-36

## Abstract

Generalized pustular psoriasis (GPP) is a rare chronic inflammatory pustular dermatosis that presents as painful erythema with sterile pustules on nonacral skin. No unified standard and guideline for the treatment of GPP has been established. Several biologics have been tried for GPP, with varying success. Acrodermatitis continua of Hallopeau (ACH) is a very rare disabling variant of pustular psoriasis characterized by sterile pustules on the fingers and toes, including the nail bed. Comparatively, treating ACH is highly challenging due to its commonly therapy-resistant disease course. The pathogenic role of IL-36 signaling axis has been currently identified in GPP development. Spesolimab, the first anti-interleukin-36 receptor biologic, has been approved for treating GPP flares and shown promising results. In view of a shared pathogenesis between GPP and ACH, specolimab may be an effective treatment for ACH. Currently, there is no case and clinical trial data exist on this condition. Therefore, this case was aim to describe real-world experience of spesolimab use in ACH coexisting with GPP. We report an Asian patient with a 16-year-history of GPP and ACH with marked pustulosis on the nail bed and onychodystrophy. He received conventional systemic regimen acitretin, cyclosporine and biologics adalimumab and secukinumab, but experienced relapse for skin lesions and refractory for nail lesions. He was then treated with a single dose of spesolimab in combination with secukinumab, which resulted in skin clearance and nearly complete resolution of nail lesions over a 32-week period. Our observation suggests that spesolimab should be considered for the treatment of ACH, especially in the patients with intractable nail lesions and concomitant GPP.

## Introduction

Generalized pustular psoriasis (GPP) is a rare, chronic, and recurring condition characterized by painful erythema and sterile pustules on nonacral skin (1). It is commonly accompanied by systemic symptoms such as fever and malaise, with severe flares potentially leading to mortality. Acrodermatitis continua of Hallopeau (ACH) is an exceptionally rare and debilitating variant of pustular psoriasis, characterized by sterile pustules on the fingers, toes, and even the nail bed, along with erythematous atrophic skin (2). ACH may precede, coincide with, or follow the onset of GPP in some cases (3, 4).

Given that both GPP and ACH are chronic and incurable conditions, achieving long-term therapeutic control becomes crucial to prevent complications. At present, there is a lack of standardized treatment guidelines for both ACH and GPP. Various biologics have been attempted for GPP, but their success rates have been inconsistent (5–7). On the other hand, managing ACH poses significant challenges as it commonly exhibits resistance to therapy. Notably, even within the same patient, the treatment response of ACH tends to be inferior to that of GPP (3, 8).

Recent studies have indicated that innate immune inflammation primarily underlies the pathogenesis of GPP, with a crucial role played by the activation of the IL-36 signaling axis (9, 10). Overactivation of IL-36 signaling and dysregulation of inflammatory cytokines released by keratinocytes contribute to the recruitment of neutrophils in the epidermis (11). Notably, mutations in the IL-36RN gene, responsible for encoding the IL-36RA, are commonly observed in both GPP and ACH patients (12, 13). Furthermore, a few GPP patients have also been found to have variants in AP1S3, a gene encoding the adaptor-related protein complex 1, sigma-3 subunit, leading to hyperactivation of tumor necrosis factor (TNF) and increased IL36α expression (14, 15). Interestingly, these variants are also observed in some ACH patients (13, 16), suggesting a shared pathogenesis between GPP and ACH, with the IL-36 signaling pathway being a potential therapeutic target. The recent approval of spesolimab, the first anti-interleukin-36 receptor (IL-36R) biologic, for the treatment of GPP flares in 2022 has shown promising results (17, 18). In this context, we present a case study of a Chinese male with concurrent ACH and GPP, who exhibited successful treatment outcomes with spesolimab after prior treatment failures with adalimumab and secukinumab.

## Case presentation

A 22-year-old Chinese male with a 7-year smoking history was referred to our department due to recurring episodes of generalized bright erythema and pustules on the trunk, limbs, fingers, toes, and nails that had been persistent for 16 years. The patient was initially diagnosed with GPP when he experienced his first flare at the age of 6. While the application of acitretin resolved the skin lesions, there was limited or no improvement in the lesions on the distal fingertips, toes, and nails. Based on the repeated pattern of relapse and remission of the finger, toe, and nail lesions, a clinical diagnosis of ACH was suspected. Histological examination of a skin biopsy from the abdomen revealed an accumulation of neutrophils in the epidermis and a moderate perivascular lymphocytic infiltrate in the dermis. The patient was administered acitretin at a daily dose of 40mg, in combination with cyclosporine at 125mg twice daily (4 mg/kg) for 24 weeks, which resulted in a slight improvement in the nail lesions. However, due to intolerable mucosal dryness, the patient discontinued the use of acitretin and cyclosporine on his own.

**Figure 1:**
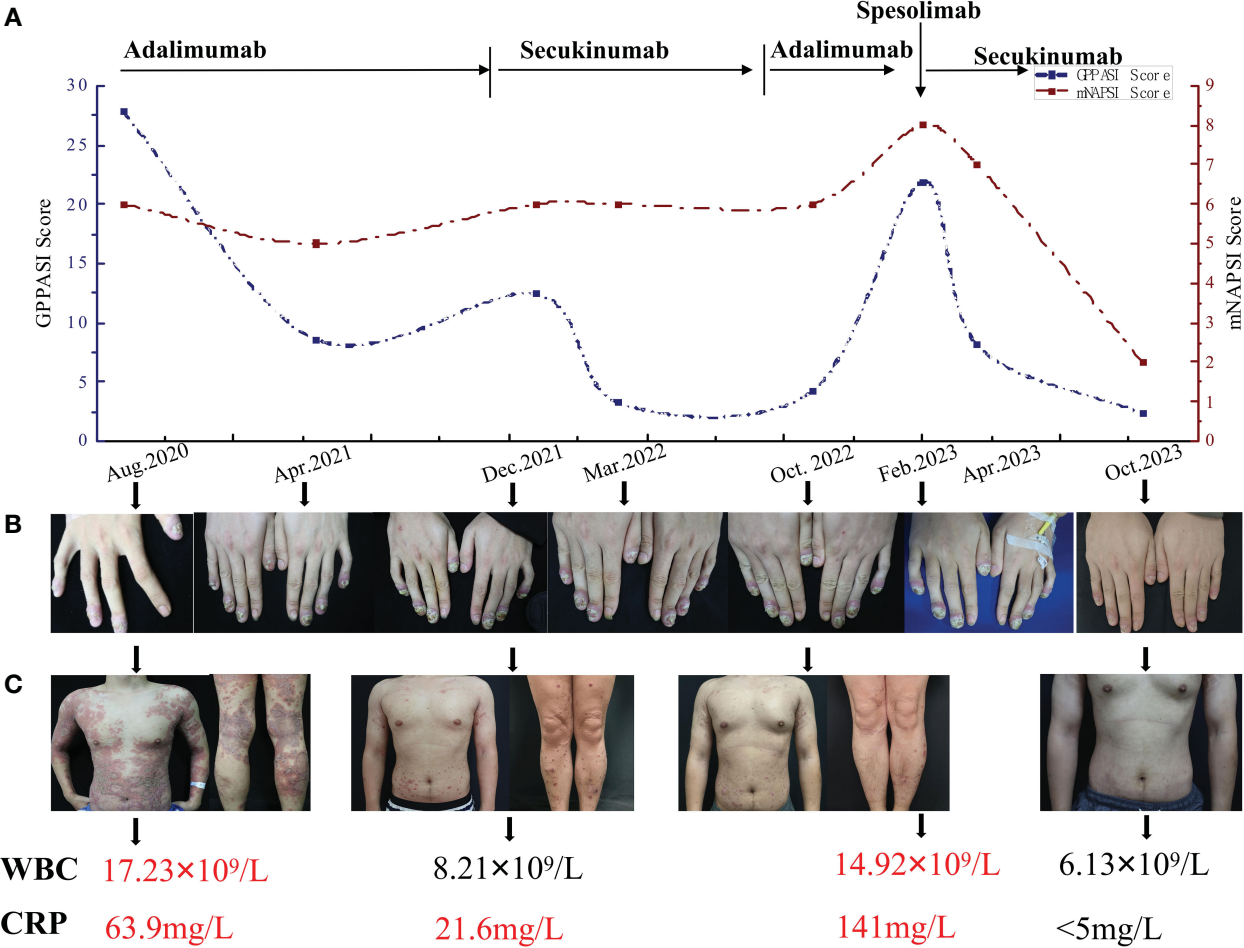
**(A)** The timeline of the GPPASI score and mNAPSI score during treatment with different biologics. **(B)** Periungual erythema, periungual and subungual pustules and onychodystrophy on swollen distal fingers in different periods. **(C)** Skin lesions on the trunk and both lower limbs and variation in the WBC and CRP. GPPASI, generalized pustular psoriasis area and severity index; mNAPSI, modified nail psoriasis severity index; WBC, white blood cells; CRP, C-reactive protein.

In August 2020, the patient was hospitalized with a diagnosis of GPP, as generalized pustules reappeared accompanied by leucocytosis and elevated C-reactive protein (CRP) levels (**Figures 1A–D**). A loading dose of 80mg of adalimumab was prescribed, followed by a subsequent dose of 40mg every 4 weeks, which led to a significant improvement in skin lesions within 3 weeks. Despite occasional recurrences of the psoriasis rash due to stress or respiratory infection during 15 months of treatment, the nail lesions persisted (**Figure 1B**). Adalimumab was then discontinued, and the patient’s treatment was switched to a loading dose of 300 mg of secukinumab every week for 5 weeks, followed by a monthly dose of 150 mg, in accordance with the Chinese guidelines for the use of biologic agents in the treatment of psoriasis (19). Improvement was observed 2 weeks after the initial injection, and after three months of treatment, the skin lesions were nearly completely resolved (**Figure 1C**). However, the patient’s nail lesions remained unresponsive to treatment. As a result, secukinumab treatment was discontinued six months later due to its high cost, and the patient was switched back to adalimumab. Within 2 months, he experienced a worsening of the skin lesions and was transferred to our hospital.

**Figure 2:**
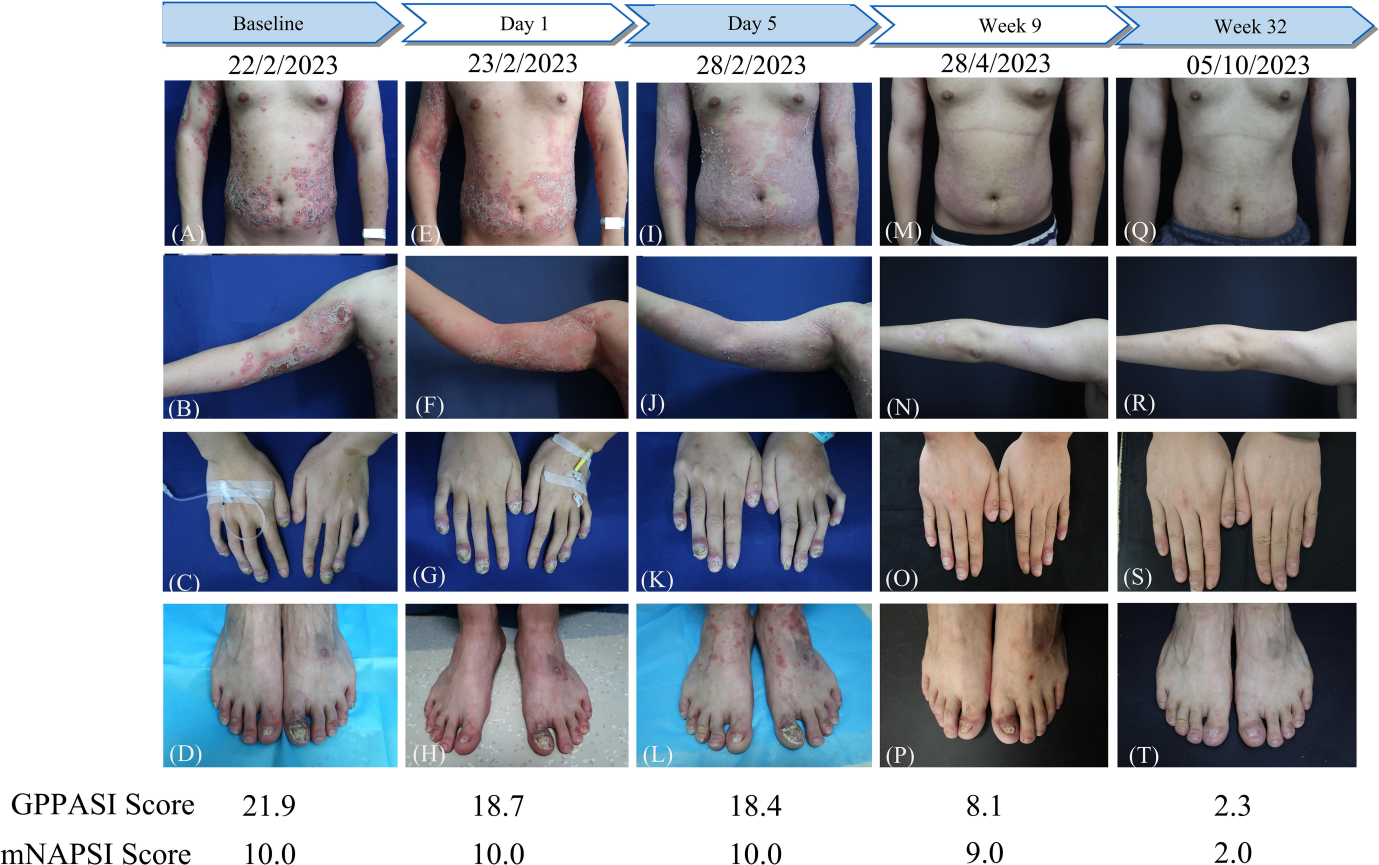
The flowchart of the patient before and after a single dose of spesolimab treatment and GPPASI and mNAPSI scores at baseline, Day 1, Day 5, week 9 and week 32. **(A-D)** Clinical appearance of the trunk, right arm, hands and toes before treatment; **(E-H)** Clearance of existing pustules and enlarged erythema on the trunk and right arm on Day 1 after treatment; clinical appearance 5 days **(I-L)**, 9 weeks **(M-P)**, and 32 weeks **(Q-T)** After treatment with spesolimab.

The physical examination revealed severe erythema accompanied by mild scales on the scalp, trunk, and upper limbs. Pustules and areas filled with pus were clearly visible at the center of several erythematous areas. Distinct demarcation lines could be observed along with erythema and pustules on most swollen distal fingers and the first toes. These areas also had erosions that were covered in thick yellow crusts. The nails on these affected digits showed signs of dystrophy and were adorned with pustules (**Figures 2A–D**). The patient’s generalized pustular psoriasis area and severity index (GPPASI) and modified nail psoriasis severity index (mNAPSI) scores were 21.9 and 10, respectively. Laboratory investigations indicated leukocytosis with neutrophilia, as well as elevated levels of CRP (**Figure 1**). Notably, the patient tested negative for rheumatoid factor, antinuclear antibody, and anti-cyclic citrullinated peptide.

In light of the notable resistance to treatment, particularly in the context of the finger and toe nails, a single intravenous dose of 900 mg of spesolimab was administered. The patient’s GPPASI and mNAPSI scores were evaluated on Day 1 (**Figures 2E–H**), Day 5 (**Figures 2I–L**), week 9 (**Figures 2M–P**), and week 32 (**Figures 2Q–T**). Following the infusion of spesolimab, pustules were significantly alleviated on the trunk and upper limbs. However, within 24 hours, several new erythematous patches emerged, extending to the patient’s forearms and a substantial portion of their trunk (**Figures 2E, F**). By Day 5, the erythema had darkened on the trunk while subsiding on the upper limbs, leaving behind scaling (**Figures 2I, J**), with no observed changes in the nail lesions (**Figures 2K, L**). After a month of spesolimab treatment, the erythema on the patient’s arm faded, and no new lesions manifested. However, erythema and pustules persisted on the distal fingers and the first toes. Consequently, the patient was prescribed secukinumab (150 mg every 4 weeks) at a local hospital. At week 9, a remarkable improvement was witnessed, with a 63% reduction in the GPPASI score (**Figures 2M, N**). Periungual inflammation diminished, and all affected fingernails began to regenerate (**Figures 2O–P**). After 32 weeks of spesolimab use, the patient’s skin and nail lesions had largely disappeared (**Figures 2Q–T**). Their GPPASI and mNAPSI scores were reduced by approximately 90% and 80%, respectively. Laboratory findings conducted at week 32 post-treatment revealed no abnormalities, and there were no reported adverse effects.

## Discussion

ACH and GPP commonly manifest as chronic and recurrent conditions that often do not respond well to conventional treatment approaches, such as acitretin, methotrexate, and cyclosporine. Biologic agents, initially used for plaque psoriasis and psoriatic arthritis (PsA), have been extensively explored as potential treatments for GPP and have shown greater efficacy compared to non-biologic systemic agents (20). Nonetheless, it is important to note that the use of TNF-α and IL-17 inhibitors has been associated with paradoxical flares of GPP and the development of new pustular lesions in some cases (21–23).

In limited case reports and small case series, encouraging effects have been observed with TNF-α and IL-17 inhibitors in managing nail lesions for ACH. Studies have shown complete resolution of nail changes within different time frames ranging from 12 to 55 weeks (24–26). It is important to note that the development of anti-drug antibody (ADA) is a significant factor contributing to treatment failure with TNF-α inhibitors (27–29). Additionally, the treatment with IL-17 inhibitors has been associated with the emergence of psoriasiform eruptions involving the fingers and nails (23), and there have been reported cases where no significant improvement was observed even after 4-8 months of therapy (30). This underscores the complex relationship of cytokines like TNF-α and IL-17 in the pathogenesis of different psoriasis subtypes, with their dominant role in plaque psoriasis not necessarily reflected in pustular psoriasis.

The IL-36 signaling pathway has emerged as a key player in the pathogenesis of GPP. Upregulation of the IL-36 cascade induces the proliferation of IL-17 and CD4^+^ Th17 cells, which in turn stimulates the expression of IL-36 and other cytokines, thereby amplifying the inflammatory response in GPP (31, 32). Numerous studies have highlighted the presence of IL-36RN mutations in patients with GPP and concomitant ACH, and loss of IL-36RN function leads to unregulated IL-36 activity (9, 12, 33). Spesolimab, targeting IL-36R ^2^, effectively inhibits the activation of pathogenic IL-36 pathways and downstream inflammasome pathways. Clinical trials have confirmed the efficacy and safety of spesolimab in GPP (17, 34). The phase 1 proof-of-concept Effisayil™ 1 study demonstrated that a single dose of spesolimab at 900 mg significantly reduces GPP severity over 20 weeks (34). Additionally, the phase 2 randomized trial revealed rapid pustular and skin clearance within one week of spesolimab treatment, with an acceptable safety profile (17). Furthermore, the Effisayil™ 2 study evaluated the efficacy and safety of spesolimab in preventing GPP flares, and recent results indicate that subcutaneous administration of spesolimab (600 mg loading dose, followed by 300 mg every 4 weeks) effectively prevents GPP flares and reduces the risk of recurrence over 48 weeks (35). Currently, phase II and III trials of spesolimab are underway in palmoplantar pustulosis, atopic dermatitis (AD), and ulcerative colitis (36–39).

To objectively assess the effectiveness of spesolimab in treating GPP lesions, we employed the GPPASI score in this particular patient. The GPPASI score is a physician-based evaluation of the severity of pustules, erythema, and scaling, with scores ranging from 0 to 72 (40, 41). Consistent with previous studies exploring the use of spesolimab in GPP treatment, a rapid clinical response was observed, with near-complete clearance of pustules within 24 hours of treatment. On Day 5, the patient demonstrated a 16.0% improvement in the GPPASI score, which falls below the previously reported range of 47.2-83.5% improvement seen one week after administration (18). Nonetheless, the patient’s skin lesions improved over the following week, eliminating the need for a second injection. The GPPASI score exhibited a remarkable decrease at week 9 and maintained this improvement until week 32.

The mNAPSI score was a reliable tool to evaluate the psoriatic nail disease in the current patient (42, 43). Since nails take 5-7 months to complete a growth cycle, assessing the efficacy of treatment for nail lesions requires a longer follow-up period compared to skin lesions (44). After 32 weeks, the patient exhibited significant improvement in nail lesions, as indicated by a 75% reduction in the mNAPSI score compared to baseline. This level of improvement had not been achieved with any previous treatments. Given the lack of response to secukinumab therapy over nearly a year, the credit for this improvement can be attributed to spesolimab rather than secukinumab. This finding suggests that IL-36 receptor antagonists hold promise as a potential therapeutic approach for the treatment of ACH.

Several limitations of the present study should be acknowledged. Firstly, a larger number of cases and longer-term follow-up are required to accurately evaluate the efficacy of spesolimab in ACH. Furthermore, it is important to recognize that concurrent PsA is estimated to be present in approximately 20.5% of ACH patients (45). Another limitation of this study was the lack of assessment of joint injuries in the patient during treatment. It is worth noting the significance of evaluating the effectiveness of spesolimab in ACH patients with concurrent joint involvement.

## Conclusion

Despite numerous proposed treatment strategies, effectively managing ACH and GPP remains a significant challenge. Given the pivotal role played by the IL-36 signaling pathway in GPP’s pathogenesis, we administered spesolimab, an anti-IL-36R biologic, to our patient. Not only did we witness a rapid regression of skin lesions, but more significantly, we achieved outstanding therapeutic outcomes for the nail lesions. To the best of our knowledge, this is the first documented case where spesolimab has been successfully employed in the treatment of coexisting ACH and GPP. Our observation strongly suggests that considering spesolimab as a treatment option for ACH, particularly in patients with challenging nail lesions in conjunction with GPP, is warranted.

## Data availability statement

The original contributions presented in the study are included in the article/supplementary material. Further inquiries can be directed to the corresponding authors.

## Ethics statement

The studies involving humans were approved by Ethics Committee on Clinical Trial, West China Hospital of Sichuan University. The studies were conducted in accordance with the local legislation and institutional requirements. The participants provided their written informed consent to participate in this study.

Written informed consent was obtained from the individual(s) for the publication of any potentially identifiable images or data included in this article.

## Author contributions

PFW: Writing – original draft, Data curation, Methodology, Visualization. CL: Writing – original draft, Data curation, Investigation. TW: Writing – review & editing. XJ: Writing – review & editing, Supervision. PW: Supervision, Writing – review & editing, Resources. SW: Supervision, Writing – review & editing, Validation.
